# Run-on mutation in the PAX6 gene and chorioretinal degeneration in autosomal dominant aniridia

**Published:** 2011-05-07

**Authors:** Shagun Aggarwal, Worapoj Jinda, Chanin Limwongse, La-ongsri Atchaneeyasakul, Shubha R. Phadke

**Affiliations:** 1 Department of Medical Genetics, Sanjay Gandhi Postgraduate Institute of Medical Sciences, Lucknow, India; 2 Department of Research and Development, Faculty of Medicine, Siriraj Hospital Mahidol University, Bangkok, Thailand; 3 Department of Ophthalmology, Faculty of Medicine, Siriraj Hospital Mahidol University, Bangkok, Thailand

## Abstract

**Purpose:**

To identify the causative paired box 6 (*PAX6*) mutation in a family with autosomal dominant aniridia.

**Methods:**

A family with autosomal dominant aniridia with three affected individuals in two generations was investigated for the causative *PAX6* mutation by single strand conformation polymorphism (SSCP) followed by sequencing of genomic DNA from peripheral blood.

**Results:**

A novel *PAX6* mutation in the donor splice site of intron 12 was identified in all three affected individuals from the family. The automated splice site analysis web interface indicated a disturbance of splicing and it was predicted that this mutation could lead to an elimination of the normal stop codon and an abnormal 3′ elongation of the mRNA.

**Conclusions:**

We report a novel *PAX6* mutation in autosomal dominant aniridia that presumably affects splicing. The presence of chorioretinal degeneration in one of the affected individual raises the possibility that run-on mutations are associated with chorioretinal involvement in aniridia.

## Introduction

Aniridia (OMIM 106210) is a panocular disorder characterized by variable degrees of deficiency of the iris tissue. In 70% cases it presents as a familial disorder with autosomal dominant inheritance. The paired box 6 (*PAX6*) gene is the only gene in which causative mutations have been identified [1,2]. Mutations leading to complete absence of the PAX6 protein are responsible for aniridia phenotype whereas milder mutations with protein production are associated with other ocular presentations like corectopia, Reiger anomaly, cataract, glaucoma, etc [3-5]. Until date, 718 sequence variants of *PAX6* and 321 pathogenic mutations have been recorded [6]. In the present paper we report the finding of a novel *PAX6* mutation in an Indian family with aniridia. We also attempt to correlate the rare occurrence of chorioretinal degeneration in one of the affected individual with the type of mutation.

## Methods

### Case description

A family with an affected father and his two affected sons had attended the genetics department of a tertiary care hospital in North India. The family hailed from the local population and had no other similarly affected family member in the previous generations.

The proband was the elder son, who was 21 years at the time of workup. He had complete absence of iris tissue, microcornea, anterior polar cataract, glaucoma, and immature cortical cataract in both eyes. In addition he had chorioretinal degeneration and multiple vitreous opacities in the right eye and retinal pigment epithelium depigmentation changes in the left eye. However his vision was relatively well conserved and he could carry out his daily chores with the aid of corrective glasses. He had undergone corrective surgery for glaucoma in both eyes 4 years ago. His intra-ocular pressure which was 43 mm-45 mm in both eyes was reduced to 14 mm after the surgery.

His younger brother who was 18 years old was legally blind since the age of 8 years. He also had complete absence of iris tissue in both eyes. In addition, he also had bilateral microcornea and opacification of both corneas more on the left side. He had glaucoma in the right eye (Intra-ocular pressure 28 mmHg) and cataract in the left eye. The left eye was diagnosed as having developed phthisis bulbi. These asymmetric eye findings in this patient could be attributed to previous injury to the left eye at the age of 14.

**Figure 1:**
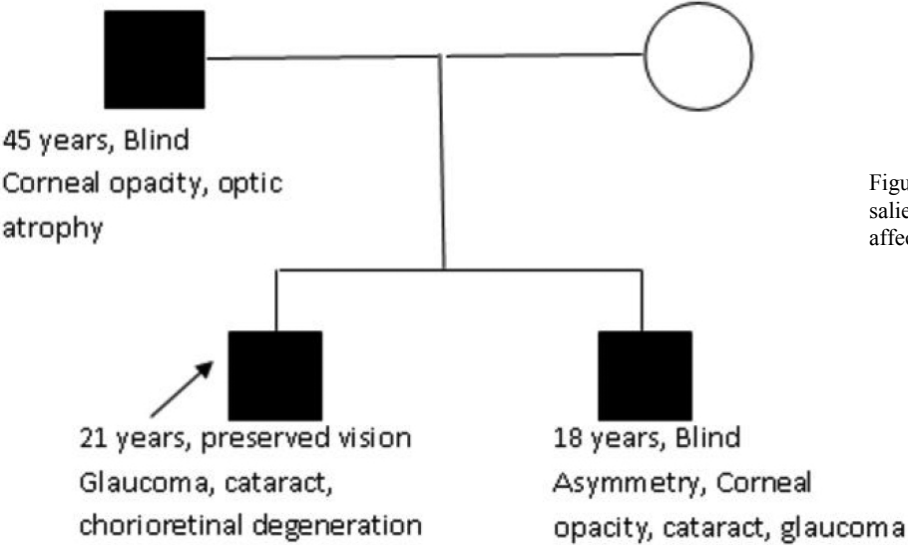
The family pedigree with salient phenotypic features of the three affected members.

The father was 45 years old and was legally blind. He had progressive visual loss since age of 25 years. On examination, he also had absence of iris tissue in both eyes. He had bilateral corneal opacities and marked thinning of the cornea. He also had optic nerve atrophy in bilateral eyes, probably due to long standing glaucoma but no prior medical records were available. The pedigree is depicted in Figure 1.

### Mutation analysis

Mutation analysis was done at the Department of Research and Development, Faculty of Medicine, Siriraj Hospital Mahidol University, Bangkok. Mutation screening in 14 exons of *PAX6* was performed using single strand conformation polymorphism (SSCP) followed by direct DNA sequencing. The primers used are as mentioned by Atchaneeyasakul et al. [7] and shown in Table 1.

**Table 1:** Depicting the primers used in the present study for amplification of *PAX6*.

**Exon**	**5'→3'**	**mer**	**Ta (°C)**	**PCR product size (bp)**
1F	GTTTGAAAAGGGAACCGTGG	20	61	243
1R	CGGCTGGAGAGTGAGAGATA	20		
2F	GCATAGGTGTGCTGGCTGCAG	21	62	350
2R	GGAGACCTGTCTGAATATTGC	21		
3F	TCAGAGAGCCCATGGACGTA	20	61	193
3R	CTGTTTGTGGGTTTTGAGCC	20		
4F	CCAGGTCCACCTCGGTTGGGA	21	67	208
4R	TGTGTCCTCCCCTGACCCTC	20		
5F	CCTCTTCACTCTGCTCTCTT	20	60	288
5R	CATAATTAGCATCGTTTACAGTAA	24		
5AF	TGAAAGTATCATCATATTTGTAG	23	57	236
5AR	GGGAAGTGGACAGAAAACCACA	22		
6F	GTGGTTTTCTGTCCACTTCC	20	60	299
6R	AGGAGAGAGCATTGGGCTTA	20		
7F	CAGGAGACACTACCATTTGG	20	60	252
7R	ATGCACATATGGAGAGCTGC	20		
8F	GGGAATGTTTTGGTGAGGCT	20	60	371
8R	CAAAGGGCCCTGGCTAAATT	20		
9F	ACAGTTTGGTCAACATATTTTG	22	60	217
9R	CAAGCACCTCTGTCTCTAGG	20		
10F	GTAGACACAGTGCTAACCTG	20	60	243
10R	CCCGGAGCAAACAGGTTTAA	20		
11F	TTAAACCTGTTTGCTCCGGG	20	63	240
11R	CCCTGAGCCACTCCTCAC	18		
12F	GCTGTGTGATGTGTTCCTCA	20	63	228
12R	TGCAGCCTGCAGAAAGCAGTG	21		
13F	CATGTCTGTTTCTCAAAGGGA	21	61	228
13R	TCCTGAAAGCTCAACTGTTGTG	22		

PCR conditions: 95 °C×7 min, 95 °C×30 s, Ta °C×30 s, 72 °C×30 s (45 cycles of step 2,3,4), 72 °C×10 min. MgCl_2_ concentration (mM) for PCR reaction of each exon: mM MgCl_2_: exon 2; 1.5 mM MgCl_2_: exon 1, 3, 4, 5FR, 6, 7, 8, 9, 10, 11, 12, 13; 2.0 mM MgCl_2_: exon 5AFAR.

## Results

**Figure 2:**
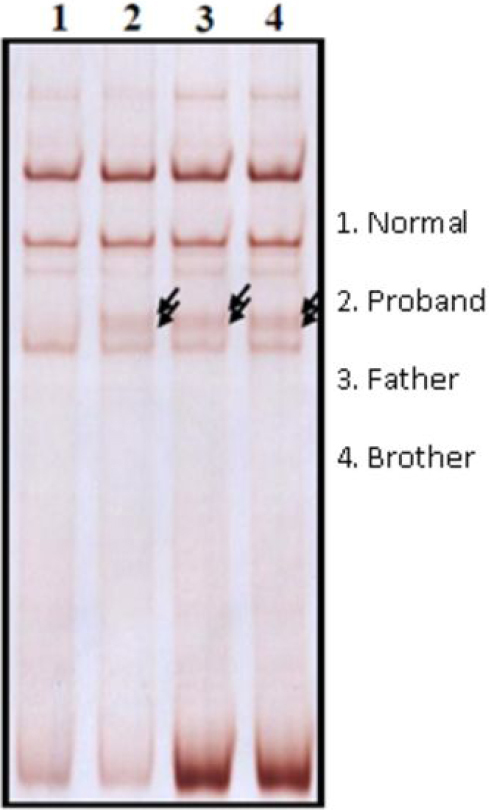
Results of SSCP analysis showing the aberrant migration of the amplification product of the mutant exon 12 in the three affected members (Lane 2, 3, and 4).

**Figure 3:**
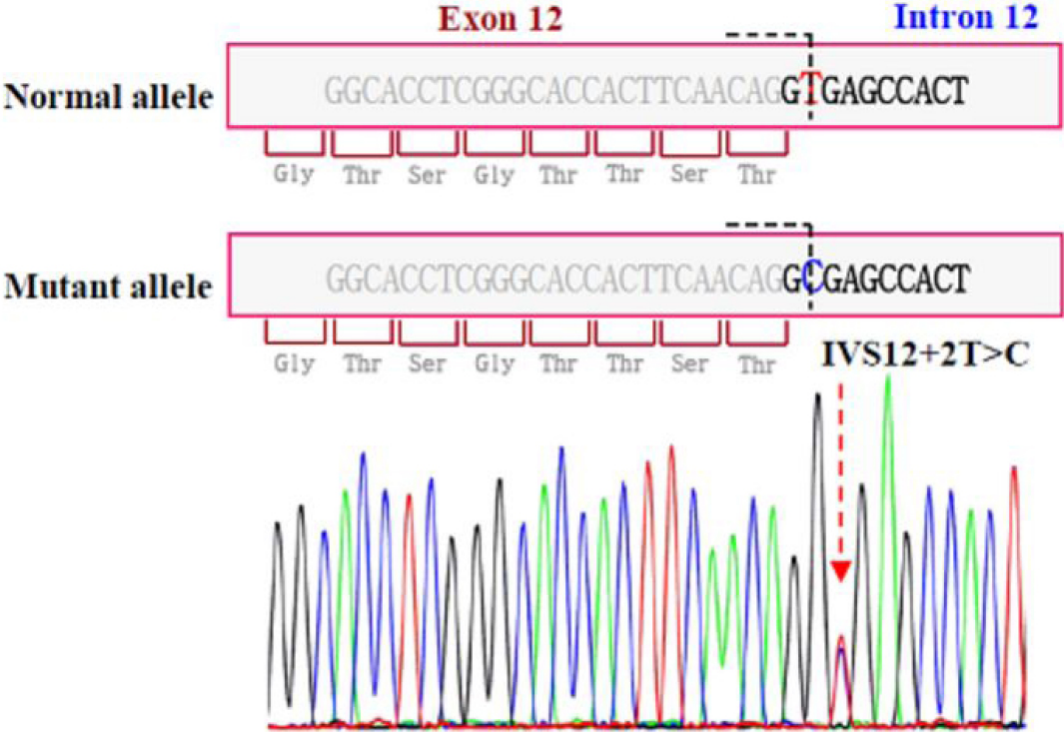
Sequencing results of Exon 12 in affected individuals showing the T→C change at donor splice site of intron 12 (IVS12+2 T>C mutation).

A heterozygous nucleotide substitution at the splice donor site of intron 12 (IVS12+2T>C) of *PAX6* was identified in the DNA samples from all three affected individuals. Screening for IVS12+2T>C in 200 normal chromosomes using SSCP analysis revealed absence of this variant. The results of SSCP analysis in the shifted fragment (exon 12) and DNA sequence segment containing IVS12+2T>C are shown in Figure 2 and Figure 3, respectively. This variant has been submitted to the Human PAX6 Allelic Variant database (DB-ID PAX6_00580) [6].

## Discussion

Aniridia is an autosomal dominant disorder resulting almost exclusively from mutations in the *PAX6* gene. It is familial in 70% cases and sporadic in others. Besides absence of iris tissue it encompasses several ocular abnormalities like glaucoma, corneal opacification, Reiger anomaly, optic nerve defects, CNS abnormalities, etc [1,2,5]. *PAX6* is an evolutionary conserved transcriptional regulator with expression in the iris tissue as well as other ocular tissues derived from neural crest cells. Its mutations are responsible for a variety of ocular phenotypes of varying severity [5,8]. Mutations resulting in introduction of premature termination codons (PTCs) account for 77% aniridia patients, these arising by nonsense mutations, splice site mutations or insertion/deletions. The remaining mutations are missense or run-on mutations [5,9]. The aniridia phenotype results from haploinsufficiency, which has been shown to arise in majority of cases by nonsense mediated decay of premRNA with premature termination codons (PTCs) [5,9]. Mutations leading to milder phenotypes are usually missense with production of aberrant but functional protein [5,9].

The mutation found in the family reported here is a novel substitution in donor splice site of Intron 12. The 10–13 exons of *PAX6* code for the PST domain, a region rich in proline, serine and threonine which functions as a transactivating domain that regulates the PAX6 protein homeodomain interaction with DNA [5,8].

The RNA surveillance mechanism cannot detect PTC in the exon13 and last 50 bp of exon 12 [5,10]. All mutations found in this region as well as splice site mutations in intron 12 are run-on mutations leading to haploinsufficiency of PAX6 by production of a protein with an elongated PST domain that interferes with homeodomain binding to DNA. Until date 6 variants in intron 12 have been found, 2 being non pathogenic and 4 associated with aniridia phenotype. One of these variants has been demonstrated to be associated with abnormally extended 3′ end of the protein, the other three not being characterized at the RNA level [6].

The splice site mutation found in this family is likely to be pathogenic by leading to aberrant splicing, but its effect on the RNA has not been established. We used the automated splice site analysis web interface to ascertain the effect of this mutation. The analyses predicted that the mutation would cause decrease in binding affinity of spliceosome proteins to this donor splice site, leading possibly to aberrant splicing [11]. This would probably lead to abolishing of the normal stop codon and abnormal 3′ elongation of the mRNA, which is the most common mechanism of pathogenicity of mutations in region distal to the 1,495th base pair of exon 12. Thus this is likely to be a pathogenic mutation in this family.

The phenotype of this family is similar to the aniridia cases where haploinsufficiency of PAX6 is the causative mechanism. However, an interesting finding is the presence of diffuse chorioretinal degeneration changes and vitreous involvement in the proband in this family. Hingorani et al. have reported the finding of Coats’ disease like exudative vascular retinopathy in two of their patients who also had run-on mutations leading to an elongated PST domain. These two patients had frameshifting mutations in the exon 13 [12]. This raises the possibility that run-on mutations leading to an abnormal carboxy terminal extension of the PST domain may be associated with chorioretinal involvement in patients with aniridia.

To conclude, we report a novel splice site mutation in *PAX6* in a family with autosomal dominant aniridia. The finding of chorioretinal degeneration in association with this probable run-on mutation indicates the possibility of a genotype-phenotype correlation.

## References

[1] Glaser T, Walton DS, Maas RL (1992). Genomic structure, evolutionary conservation and aniridia mutations in the human PAX6 gene.. Nat Genet.

[2] Hanson IM, Seawright A, Hardman K, Hodgson S, Zaletayev D, Fekete G, van Heyningen V (1993). PAX6 mutations in aniridia.. Hum Mol Genet.

[3] Hanson I, Churchill A, Love J, Axton R, Moore T, Clarke M, Meire F, van Heyningen V (1999). Missense mutations in the most ancient residues of the PAX6 paired domain underlie a spectrum of human congenital eye malformations.. Hum Mol Genet.

[4] Hanson IM, Fletcher JM, Jordan T, Brown A, Taylor D, Adams RJ, Punnett HH, van Heyningen V (1994). Mutations at the PAX6 locus are found in heterogeneous anterior segment malformations including Peters' anomaly.. Nat Genet.

[5] Tzoulaki I, White IM, Hanson IM (2005). PAX6 mutations: genotype phenotype correlations.. BMC Genet.

[6] The PAX6 Allelic Variant Database http://pax6.hgu.mrc.ac.uk/

[7] Atchaneeyasakul LO, Trinavarat A, Dulayajinda D, Kumpornsin K, Thongnoppakhun W, Yenchitsomanus PT, Limwongse C (2006). Novel and de-novo truncating PAX6 mutations and ocular phenotypes in Thai aniridia patients.. Ophthalmic Genet.

[8] van Heyningen V, Williamson KA (2002). PAX6 in sensory development.. Hum Mol Genet.

[9] Vincent MC Pujo AL Olivier D Calvas P Screening for PAX6 gene mutations is consistent with haploinsufficiency as the main mechanism leading to various ocular defects. Eur J Hum Genet 2003 11 163 9 12634864 10.1038/sj.ejhg.5200940

[10] Byers PH (2002). Killing the messenger: new insights into nonsense-mediated mRNA decay.. J Clin Invest.

[11] Automated splice site analyses http:\\www.splice.uwo.ca

[12] Hingorani M, Williamson KA, Moore AT, van Heyningen V (2009). Detailed ophthalmologic evaluation of 43 individuals with PAX6 mutations.. Invest Ophthalmol Vis Sci.

